# Radiooncological View on Therapy Outcome after Multidisciplinary Treatment of Sinonasal Tumors

**DOI:** 10.3390/cancers13102364

**Published:** 2021-05-14

**Authors:** Olena Klymenko, Anna Maria Stefanie Buchberger, Barbara Wollenberg, Klaus-Dietrich Wolff, Victoria Kehl, Stephanie E. Combs, Anja Pickhard, Steffi U. Pigorsch

**Affiliations:** 1 Department of Radiation Oncology, Technical University of Munich (TUM), Ismaninger Straße 22, D-81675 Munich, Germany; olena.klymenko@mri.tum.de (O.K.); stephanie.combs@tum.de (S.E.C.); 2 Ear, Nose and Throat Department, Head and Neck Surgery, Technical University of Munich (TUM), Ismaninger Straße 22, D-81675 Munich, Germany; maria.buchberger@tum.de (A.M.S.B.); barbara.wollenberg@tum.de (B.W.); 3 Department of Oral and Maxillofacial Surgery, Technical University of Munich (TUM), Ismaninger Straße 22, D-81675 Munich, Germany; klaus-dietrich.wolff@tum.de; 4 Institute of Medical Informatics, Statistics and Epidemiology, Technical University of Munich (TUM), Ismaninger Str. 22, D-81675 Munich, Germany; victoria.kehl@tum.de (V.K.); anja.pickhard@tum.de (A.P.); 5 Institute of Radiation Medicine (IRM), Helmholtz Zentrum München, Ingolstädter Landstraße 1, D-85764 Neuherberg, Germany

**Keywords:** sinonasal tumors, tomotherapy, IMRT, simultaneous integrated boost (SIB), radiochemotherapy, gross tumor volume (GTV)

## Abstract

**Simple Summary:**

Tumors of the nasal cavity and paranasal sinus are rare. Most are discovered at a locally advanced stage and require multimodal treatment consisting of surgery and radiotherapy with concomitant chemotherapy. Tumor localization between the eyes and near the brain makes treatment planning difficult due to the necessary consideration of both critical normal tissue with high protection priority and the curative aim of the best radiotherapy dose deposit. Thus, it is noteworthy how tumor volumes impact the five-year survival outcome. Therefore, we investigated initial tumor volumes before any therapy. Patients with larger mean initial tumor volumes of more than 75 cm^3^ had worse outcomes. There was no additional benefit of upfront surgery. Especially for patients with large tumor burdens of the nasal cavity or paranasal sinus, an interdisciplinary case discussion with the patient is essential in the process of shared decision-making.

**Abstract:**

Purpose: We report the outcome of a mono-institutional retrospective study of sinonasal carcinoma with the primary focus on GTV (gross tumor volume) and the effect of radiotherapy. Methods: 53 patients with sinonasal carcinoma and that of the nasal cavity, paranasal sinus or both except lymphoma were included. All patients were treated between 1999 and 2017. For tumor volume delineation, all pre-therapeutic images were fused to the planning CT (computed tomography). Results: The median follow-up was 17 months [0.3–60], the median age 60 years, 35 males and 18 females were included. Squamous cell carcinoma (SCC) (60.4%) was the predominant histology, followed by adenocarcinoma (15.1%). The mean composite OS (overall survival) time was 33.3 ± 3.5 months. There was no significant difference in the 5 y composite OS between tumor localization or radiotherapy setting. The simultaneous integrated boost concept showed a trend towards improving five-year composite OS compared to the sequential boost concept. The only factor with a significant impact on the 5 y composite OS rate was the pre-therapeutic GTV (cutoff 75 cm^3^; *p* = 0.033). The GTV ≥ 100 cm^3^ has no effect on the 5 y composite OS rate for SCC. Conclusions: The pre-therapeutic GTV is a prognostic factor for five-year composite OS for the entire group of patients with sinonasal tumors, influencing the outcome after completion of all treatment strategies. The GTV seems to not influence five-year composite OS in SCC. For this rare tumor entity, an intensive, multidisciplinary discussion is essential to finding the best treatment option for the patient.

## 1. Introduction

Sinonasal malignancies are very rare tumors, with an incidence of approximately 0.6 per 100,000 people. Only approximately 3% of all head and neck tumors are located in the paranasal sinuses or nasal cavity [1,2] and comprise only 0.2–0.3% of all cancers [3]. They are more common among males [2,4] and are most frequently found in the maxillary sinus and nasal cavity [2]. The prognosis of patients with sinonasal carcinomas, despite progress in therapy modalities and regimes, remains poor. Tumor histology, T and N stage, and age are factors that influence therapy outcome [5,6]. Five-year overall survival ranges from 30–80% [2]. The median OS time is given with 27.6–98.6 months [6,7,8,9].

Sinonasal tumors have very heterogeneous histology and are partly occupation-attributed cancer, for instance, after years of working with wood as a carpenter or in the leather manufacturing industry. Squamous cell carcinoma (SCC) (up to 70%) and adenocarcinoma (AC) are predominant, followed by adenoid cystic carcinoma (ACC), mucoepidermoid carcinoma (MC), sinonasal neuroendocrine carcinoma (SNEC), sinonasal undifferentiated carcinoma (SNUC), and esthesioneuroblastoma [4,10]. The discrepancy observed in the survival outcome could be explained by the vast heterogeneities in histological characteristics of sinonasal tumors. Surgery before radiotherapy (RT) is the standard of care for sinonasal tumors [4,11]. There are different surgical approaches for resection of sinonasal tumors, e.g., endoscopic surgery or craniofacial resection, depending on the localization of the primary. The invasion of the orbita and its structures is classified into three grades (I—destruction medial orbital wall; II—periorbital fat invasion, without invasion of the cone, III—infiltration of the medial rectus muscle, optic nerve, bulb or eyelid). One aim of surgery is the conservation of the eye. Thus only upon grade III invasion is an exenteration bulbi performed [12]. Especially after bulbus conserving surgery, the close proximity of organs at risk (OAR) of the optical system renders it difficult to deliver an appropriate radiation dose to the tumor site while minimizing the dose to the OAR to reduce the risk for late radiation toxicity. Over the last two decades, intensity-modulated radiotherapy (IMRT) has become the standard of care for head and neck cancer. Several clinical monocentric studies reported that IMRT reaches optimal dose-volume distribution in postoperative as well as in the definitive treatment of sinonasal tumors [13,14]. IMRT is applied via different radiotherapy machines, with special features depending on the used IMRT technique. One application form is IMRT via linear accelerator, in a special way as volumetric modulated arc therapy (VMAT) or via a tomotherapy machine. Some specialized facilities use protons or heavy ions and can reduce doses to OAR in a better way. Over the years, different boost RT techniques have been developed: the sequential (SEQ) and the simultaneous integrated boost (SIB) technique. The current RT strategy is to apply 70 Gy to the macroscopic tumor for definitive RT and 60 to 66 Gy to the resection bed after surgery for adjuvant RT. Especially teams treating patients with proton or heavy ions have pushed the border for steep dose gradients to surrounding normal tissue and for local tumor control. In addition, higher radiation doses compared to 3D-conformal RT can be applied by IMRT or VMAT with synchronous sparing of OAR. Many studies reported low radiation-induced toxicity after introducing IMRT to treat sinonasal tumors [13,15,16,17,18,19,20].

There are no randomized prospective trials to treat sinonasal cancer in general available. Surgery alone or in combination with radiotherapy is still the preferred treatment in most cases, depending on the tumor stage. Chemotherapy is always a particular point of discussion in the multidisciplinary concept.

The histological type was shown to predict a better outcome for low-grade AC compared to high-grade AC. Yet, the addition of RT significantly improved the outcome of patients with high-grade AC [21]. First-line surgery is the method of choice for ACC. Surgery plus RT improved local control for advanced ACC [22]. Nevertheless, the main prognostic markers in this study were the presence of distant metastasis and an advanced tumor stage.

For unresectable advanced sinonasal carcinomas, especially ACC or SCC, reports showed that definitive radiochemotherapy (RCT) applied by IMRT is a reliable treatment option [14]. Superior results can also be yielded by a combined RT of photon and protons or carbon ions in specialized centers [23]. Thus, ACC and AC have a better 5 year OS of up to 60–80%, while SNEC and mucosal melanoma have only 30–35% [2,5,22]. SCC has an intermediate 5 year OS rate of approximately 60% [24].

Adjuvant RT improves survival and locoregional control (LRC) for patients with ACC with an R1 or R2 resection status, but RT provided no further benefit after R0 resection [25]. The opposite effect was seen for mucoepidermoid carcinoma. Interestingly, Auger et al. reported no predictive impact of positive margins for the survival of mucoepidermoid carcinoma treated with adjuvant RT [26]. Contradictorily, survival benefit was demonstrated for adjuvant RT after R0-resection only. Hence, this study did not investigate the association between low and high-grade tumors and survival independent of RT. Surgery remains the treatment of choice for mucoepidermoid carcinoma as well as for mucosal melanoma [26]. Promising immunotherapy treatment results for mucosal melanoma could lead to a change [27]. For SNUC, the therapy outcome remains poor [28]. It was described that conduction of neo-adjuvant RT followed by surgery or induction chemotherapy followed by concurrent RCT in SNUC patients allows for an increase of the 2 year OS rate of up to 64% in both treatment regimens [29].

Xerostomia and dysgeusia are the most frequent late radiation side effects than changes in the sense of smell and dry nose after RT [30]. Chronic side effects influence the quality of life (QoL) of long-term survivors [30]. Before treatment decisions are made, all cases should be discussed within a multidisciplinary team to ascertain the best treatment option for the individual patient. The patient requires all information concerning treatment options and multimodal concepts as well as potential side effects of possible therapeutic approaches for the process of shared decision-making. Published data shows the multifactorial influence on the outcome after various therapy regimens. Through the present retrospective study, the outcome of patients with sinonasal tumors treated with varying radiooncological concepts was investigated at our center. Specific focus was placed on how the initial macroscopic tumor volume correlated with the composite overall survival as an effect of definitive or adjuvant radiotherapy.

## 2. Methods

### 2.1. Patient Characteristics

This study is a retrospective single institutional investigation. Patient data were retrieved from patient charts, the internal radiooncology database, interdisciplinary tumor conferences, and the Munich tumor registry. In total, we were able to identify 53 patients who were treated at the Department of Radiooncology between 1999 and 12/2017. TNM classification was done according to the 7th edition of UICC, 2010. The project was granted ethical approval by the ethics committee of the Medical Faculty of Technical University of Munich (No. 112/15 and 116/15).

### 2.2. Treatment Characteristics

Radiotherapy was delivered by 3D-conformal technique (1999–2007) and as of 2007, by IMRT using helical tomotherapy (HT) or IMRT with step and shot or sliding window and later volumetric modulated arc (VMAT) therapy as RapidArc^TM^ (RA) using a linear accelerator (Rapid Arc^TM^, Varian Medical Systems, Inc. Palo Alto, CA, USA; HI-ART TomoTherapy, Accuray, Madison, WI, USA). Patient immobilization was performed using a thermoplastic head and shoulder mask. The contrast-enhanced CT with a slice thickness of 3 mm portrayed the entire head, including the brain to the aortic arch and the middle of the mediastinum. The CT images were reconstructed with 512 × 512 pixels matrices. Every patient received a planning magnetic resonance imaging (MRI) of the head and neck, which was coregistered to the planning CT. Target delineation after coregistration of all available images was done using iPlan^®^ software (Brainlab AG, Munich, Germany). All available pre-therapeutic images (especially before surgery) were also coregistered. For calculation of pre-therapeutic GTV, in all cases, the initial images (MRI or CT) were coregistered using iPlan^®^ software (Brainlab AG, Munich, Germany). The GTV was delineated on axial, coronal, and sagittal MRI images (T1, T2 sequence without and with contrast enhancement) and CT images and calculated in cm^3^ via software.

### 2.3. Helical Tomotherapy

Treatment planning was done with the TomoTherapy planning station (Accuray), version 2 to 4.2.3. The tomotherapy plans were all helical IMRT plans with field widths of 1 or 2.5 cm and calculation grid size set to fine.

### 2.4. VMAT and IMRT

Treatment planning for the VMAT and dynamic IMRT plans was performed with the treatment planning system (TPS) Aria Eclipse, version 8.6 to 13.0 (Varian Medical Systems, Palo Alto, CA, USA). For dose calculation, the anisotropic analytical algorithm (AAA, version 10.028) was used with a dose grid size of 2.5 × 2.5 × 2.0 mm^3^. VMAT and IMRT planning was performed for treatment using a Varian Clinac Trilogy linear accelerator equipped with a 120 HD MLC using 6 MV photons. VMAT plans usually consisted of two to three arcs with 358° rotation each. The sliding window IMRT plans were optimized using a beam set-up of seven or nine coplanar static beam directions with evenly distributed angular distances.

**Figure 1:**
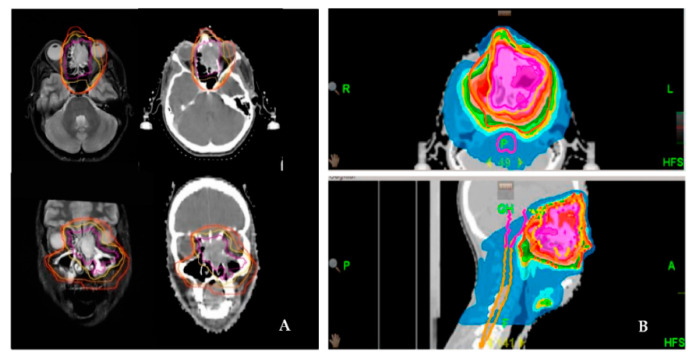
Delineation and dose distribution by helical tomotherapy of a left-sided paranasal carcinoma with orbital involvement. Definitive radiation treatment was performed using a SIB concept on Hi-Art tomotherapy (**A**). Delineation of the target volume on both CT and MRI scans (magenta: GTV; yellow: SIB-1; orange: SIB-2; red: PTV). (**B**). Dose distribution as a color wash.

For definitive RT, gross tumor volume (GTV) was defined on planning MRI and contrast-enhanced CT. For adjuvant RT planning, the preoperative and the postoperative images were also coregistered to the planning CT scan. Over the years, two different boost concepts were applied: sequential boost concept (SEQ) and simultaneous integrated boost concept (SIB). For adjuvant treatment, after delineation of the pre-surgery GTV, an isotropic margin of 5 mm for SIB 1 and 10 mm for SIB 2 and 10 mm for SQB by the TPS was deemed as the clinical target volume (CTV1 or SIB1, respectively) was generated. Manual correction on each slice fitted the CTV1/SIB1 to anatomical borders (e.g., bone). For definitive treatment in cases of SIB planning, the SIB2 volume was created by another 5 mm margin around SIB1 (Figure 1). For the planning target volume (PTV) for both radiation settings, a further 5–10 mm and a set-up error of max. 3 mm was generated and manually corrected on each slice. The cervical lymph node levels I-III and retropharyngeal nodes were irradiated only in cases of histologically positive cervical lymph nodes or with macroscopic evidence in definitive situations.

For the SEQ concept, the single dose was 1.8–2.0 Gy to 50 Gy to the PTV, and thereafter, a cumulative dose of 60–66 Gy (postoperative setting) or 66–70 Gy (definitive setting) was applied to the boost volume. The SIB concept consisted of two SIBs for definitive and of one SIB for adjuvant setting. Definitive SIB1 (GTV plus 5 mm) received a single dose of 2.2 Gy to 70.4 Gy, and an adjuvant SIB (pre-therapeutic GTV plus 10 mm) was irradiated with 2.14 Gy to 64.2 Gy. Dmean was normalized to SIB1. The definitive SIB2 (GTV plus 10 mm) was 2.0 Gy to 64 Gy. The adjuvant PTV was irradiated with a 1.8 Gy single dose to 54.0 Gy, and the definitive PTV was 1.7 Gy to 54.4 Gy.

For plan optimization, the following OARs were included: brainstem, temporal lobe, optical nerve, retina, chiasm, lenses, lacrimal gland, parotid gland, submandibular gland, inner ear, oral cavity, and mandible. In the case of proximity or overlap of an OAR (e.g., optical nerve or chiasm), an underdosage of the PTV resp. SIB was permissible for the sake of staying within the OAR limits. Thus, a maximally achievable dose to the PTV/SIB and the minimally achievable dose to the OAR within the tolerance limits were obtained.

After treatment, all patients underwent regular lifelong tumor aftercare (commencing 6 weeks post-RT) in cooperation with both the Ear, Nose and Throat Department and the Department of Oral and Maxillofacial Surgery.

### 2.5. Concurrent Chemotherapy

Chemotherapy was indicated in positive tumor resection margins and/or positive extracapsular nodal status for adjuvant treatment or in definitive settings. Concurrent chemotherapy was platinum-based. Dosage was cisplatin 40 mg/m^2^ or carboplatin AUC 2 weekly, depending on comorbidity. A maximum of 6 cycles was administered. In a few cases, cisplatin was chosen with 100 mg/m^2^ d 21 for two cycles. Until 2007, cisplatin was given in weeks 1 and 5 at 20 mg/m^2^ d1-5.

### 2.6. Statistical Methods

All statistical analyses were performed using IBM SPSS statistics software (version 24, Armonk, NY, USA: IBM Corp.). Time-to-event analysis was performed using the Kaplan–Meier method. Evaluation of differences in survival curves between various groups was performed using the log-rank test. The composite event (composite overall survival) consisted of death or recurrence or progression (whichever occurred first). The 5 year composite OS values were shown as the cumulative proportion surviving at the time (%) and the corresponding standard error. The exploratory significance level was set to 5%.

## 3. Results

Fifty-three patients were analyzed in the present study and treated at the Department of Radiooncology and Radiotherapy of the Technical University of Munich between 1/1999–12/2017. Patient characteristics are shown in Table 1. Most patients were diagnosed with locally advanced tumors of UICC-stages III–IV (83%). Squamous cell carcinoma was the most frequent tumor type, followed by adenocarcinoma, mostly located within the paranasal sinus.

30 out of 53 patients were treated after radical tumor resection using postoperative RT. 19 out of 53 patients underwent definitive RT without surgery. Two patients had an incomplete tumor resection (R2 status) and, therefore, received definitive RT. One patient suffered from early recurrence after surgery and was treated by definitive RT. Another patient had RT in palliative intention for tumor recurrence (Table 2). 23 out of 53 patients underwent concurrent chemotherapy. The course of RT lasted 43 days on average. The median follow-up was 17 months [0.3–60]. Since 2008, 49 out of 53 patients have been treated with IMRT either delivered by LINAC (*n* = 18) or by HT (*n* = 31).

### Five-Year Composite Overall Survival

**Figure 2:**
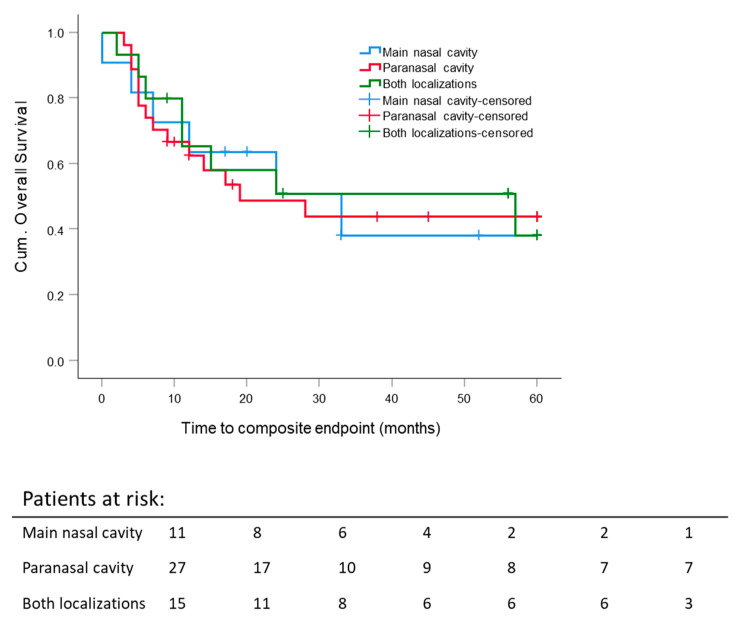
Five-year composite overall survival: All patients stratified according to the primary tumor site, such as the nasal cavity, paranasal sinuses and both localizations. Kaplan–Meier curves were calculated from the date of diagnosis.

The estimated mean composite OS time for all patients was 33.3 ± 3.5 months. There was no significant difference in the 5 year composite OS rate pertaining to differing tumor localizations (nasal cavity: 38.2 ± 16.4%, paranasal sinus: 43.8 ± 10.3%, and both (combined tumor extension) 38.2 ± 14.9%) (Figure 2).

The Kaplan–Meier analysis by tumor histology comparing SCC and “salivary gland carcinoma” as AC, ACC, and MC (esthesioneuroblastoma and SNUC were excluded from this analysis) revealed no statistically significant difference for the cumulative event-free survival.

**Figure 3:**
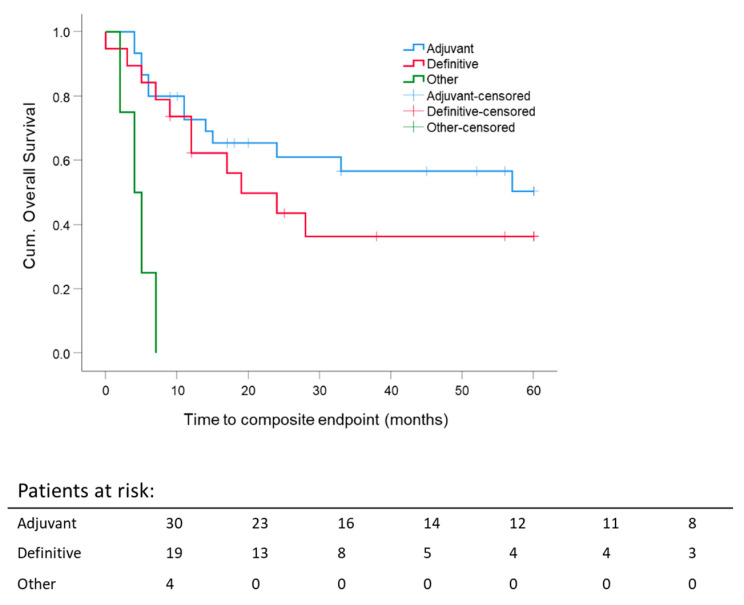
Five-year composite overall survival depending on the radiation settings: stratification for RT intention adjuvant/postoperative; definitive; others: palliative, early recurrence, additive. Kaplan–Meier curves for each analyzed group were calculated from the date of diagnosis.

By the first year after diagnosis, adjuvantly and definitively treated patients reached comparable composite OS, but the five-year composite OS calculated for different RT intentions revealed better composite OS for adjuvant RT (Figure 3). Nevertheless, 5 y composite OS after adjuvant RT was not significantly better when compared to definitive RT (adjuvant: 50.4 ± 10.4% vs. definitive 36.4 ± 12%). Survival of the remaining patients (palliative, early recurrence, and additive RT) was poor with less than 10 months.

**Figure 4:**
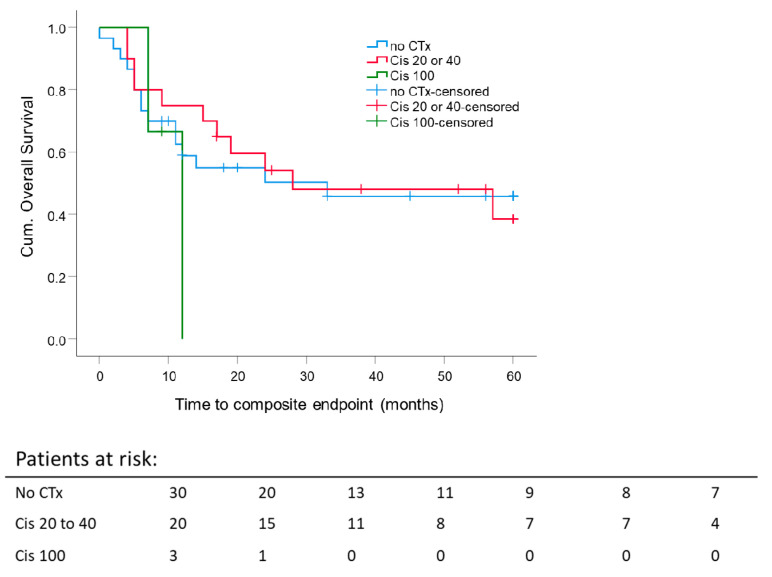
Impact of concurrent chemotherapy on 5 year composite overall survival: Kaplan–Meier curves were calculated from the date of diagnosis for 23 patients treated with concurrent chemotherapy. *p* = 0.62.

There was no impact of the chemotherapy schedule compared to patients without chemotherapy on five-year composite OS independent of RT intention. Thirty patients had only RT. Twenty patients received cisplatin 20 mg/m^2^/d at weeks 1 and 5 for five days or cisplatin 40 mg/m^2^ weekly. Three patients were treated with cisplatin 100 mg/m^2^ qd21 three times over the course of radiotherapy because of very locally advanced or systemically metastasized sinonasal tumors at the time of diagnosis. The latter showed a worse outcome (Figure 4).

5 year composite OS rate for RT alone was 45.8 ± 9.8%, for radiochemotherapy (cisplatin 20 or 40 mg/m^2^): 38.5 ± 12.6% (*p* = 0.62). Of the cisplatin 100 mg/m^2^ group, no one survived longer than 12 months.

**Figure 5:**
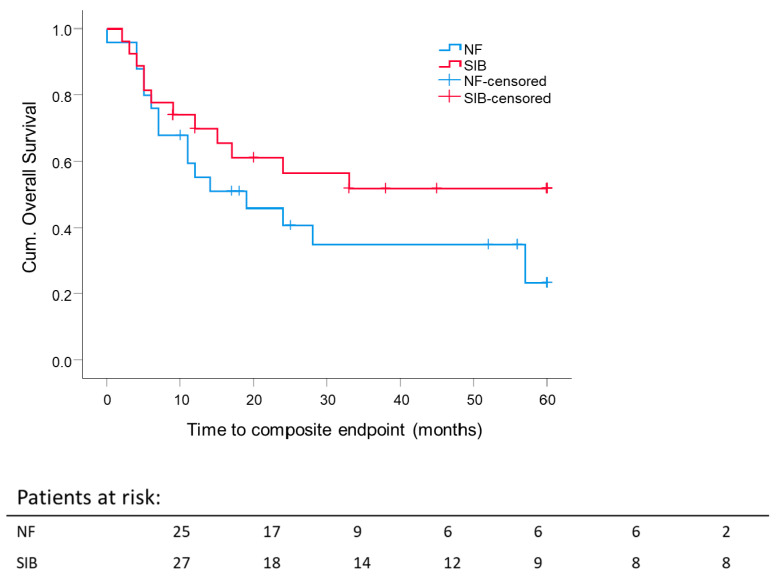
Impact of boost concept on 5 year composite overall survival: Kaplan–Meier curves for each analyzed group were calculated from the date of diagnosis. *p* = 0.177.

Data of 52 patients were available for analysis of the boost. One patient had both boost concepts and was excluded from this analysis. A comparison of the 5 year composite OS rate stratified for boost concepts showed no significant difference (SEQ: 23.3 ± 11.8% and SIB: 51.8 ± 10.2%) (Figure 5). Nevertheless, despite no significant result, we observed a clear trend towards a better composite OS after SIB. The mean composite OS for SIB was 36.9 months ± 5.0 months vs. SEQ 28 months ± 5.0 months.

For 48 out of 53 patients, the analysis of primary GTV before surgery or RT was possible. The median GTV of the entire (resected and unresected patients) cohort was 76.6 cm^3^. To investigate the effect of the median GTV on composite OS, the GTV cutoff was set at 75 cm^3^.

**Figure 6:**
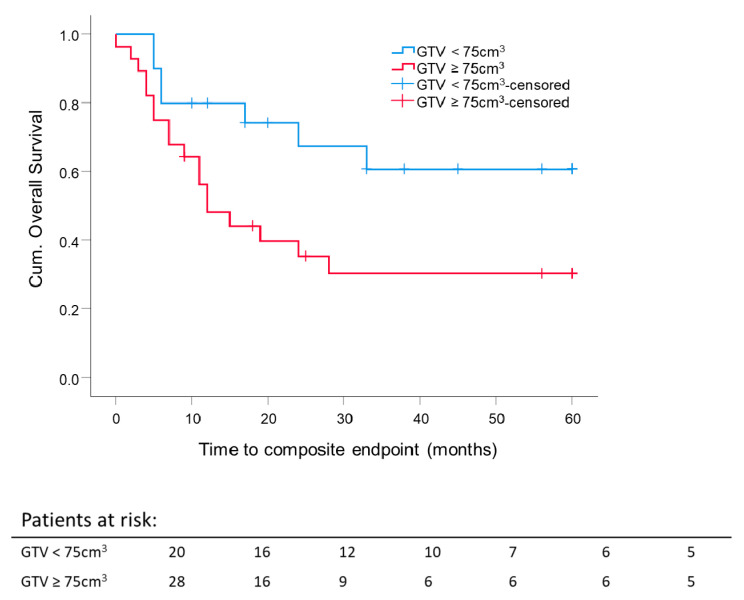
Five-year composite overall survival depending on the GTV: All patients were stratified according to initial GTV, either before surgery or before radiotherapy. Cutoff is 75 cm^3^. Kaplan–Meier curves for each analyzed group were calculated from the date of diagnosis. *p* = 0.033.

**Figure 7:**
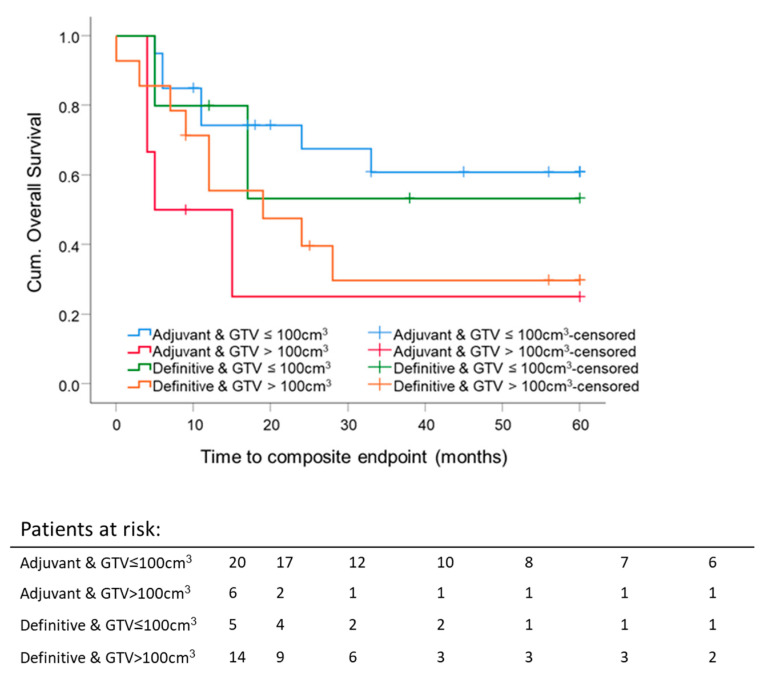
Five-year composite overall survival depending on the GTV and radiation settings: All patients were stratified according to adjuvant and definitive radiation and GTV. GTV cutoff is 100 cm^3^. Kaplan–Meier curves for each analyzed group were calculated from the date of diagnosis.

The 5 year composite OS difference depending on a GTV with 75 cm^3^ as a cutoff was significant (*p* = 0.033). Five-year composite OS for patients with GTV ≤ 75 cm^3^ was 60.8 ± 11.9%, and for the group with GTV ≥ 75 cm^3^, 30.3 ± 9.4%, independent of RT setting (Figure 6). The mean GTV of all eligible patients was 98 cm^3^. Thus, GTV at 100 cm^3^ was set as a cutoff value for further investigation. Forty-five patients were eligible for this analysis; 3 patients were excluded because of different treatment intentions (neither adjuvant nor definitive RT). For patients with large initial tumor volumes, GTV ≥ 100 cm^3^, the 5 year composite OS was lower for both definitive RT (29.8 ± 13.3%; median composite OS 18.7 months) and adjuvant RT (25 ± 20.4%; median composite OS 9.2 months) compared to smaller initial tumor volumes, GTV ≤ 100 cm^3^, for definitive RT (53.3 ± 24.8%; median composite OS 60 months) or adjuvant RT (60.9 ± 11.9%; median composite OS 60 months). For adjuvant and definitive RT, the median composite OS time for patients with tumor volumes smaller than 100 cm^3^ was 60 months. There was no significant difference in the five-year composite OS between adjuvant and definitive RT per GTV-cutoff group. The highest 5 year composite OS was reached for GTV ≤ 100 cm^3^ after surgery, followed by adjuvant RT. The worst 5 year composite OS was achieved after surgery, followed by adjuvant RT for tumors with an initial GTV ≥ 100 cm^3^ (Figure 7).

**Figure 8:**
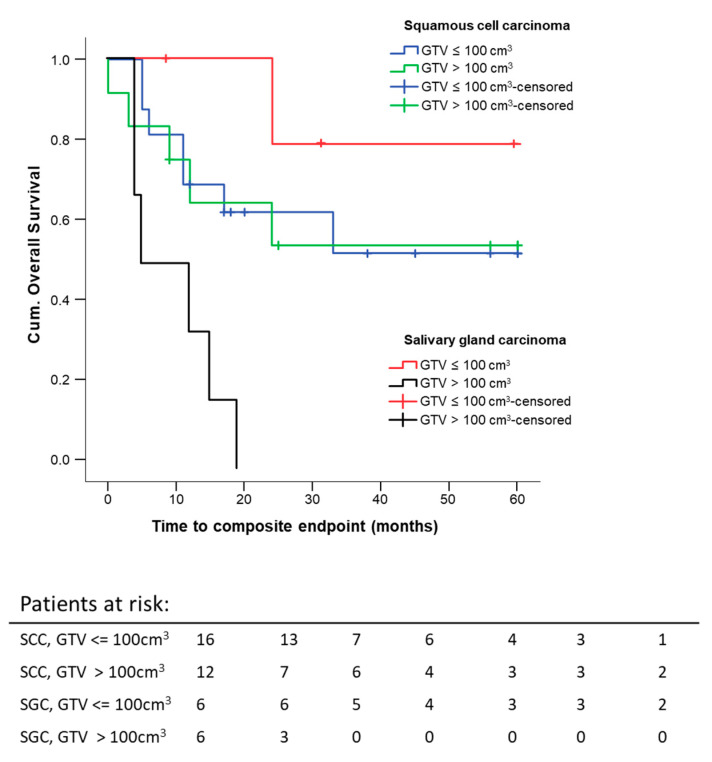
Five-year composite overall survival depending on the GTV and histology: All patients were stratified according to histology and GTV. Kaplan–Meier curves for each analyzed group were calculated from the date of diagnosis.

Histology seems to influence the effect of pre-therapeutic GTV on 5 year composite OS, but the number of patients with salivary gland histology was only *N* = 12 compared to SCC with *N* = 28. As expected, the survival curve for the salivary gland carcinoma subgroup was lower than the one for the SCC subgroup of patients. This difference was not statistically significant (*p* = 0.208), but the trend was as expected. Another effect which we saw was that tumor size did not seem to have an effect on survival in the SCC patients (*p* = 0.915), whereas large salivary gland tumors performed much worse concerning survival (*p* = 0.001) (Figure 8). The last result is based on 12 patients with 7 events, so it needs confirmation in a larger trial.

## 4. Discussion

In this mono-institutional study, we present the outcome of 53 patients with sinonasal tumors after treatment between 1999–2017. In point of fact, it was previously shown that SCC, followed by AC, is the most frequently observed histologies among sinonasal tumors [4,10], just as we found for our cohort. The mean composite OS time for the presented cohort was 33 months. Tumor location did not significantly influence the five-year composite OS rate. Treatment setting of RT (adjuvant or definitive) influences five-year composite OS, while the composite OS rate for adjuvant and definitive RT is comparable in the first posttreatment year. This differs slightly after five years, favoring the adjuvant group. The mean and the median pre-therapeutic GTV influence the five-year composite OS.

In a retrospective, mono-institutional study, Duru Birgi et al. investigated 43 patients with SCC of the nasal cavity or paranasal sinus. There was no difference for SCC concerning the outcome, neither after adjuvant nor definitive RT after two years. The overall 5 year OS rate was 71% [31]. Data from the Memorial Sloan Kettering Cancer Center (MSKCC) showed a difference in outcome for adjuvant and definitive RT. The subgroup of SCC had a worse 5 year OS of 15% after RT than the 67% after surgery followed by RT [13,15]. As a significant prognostic factor for outcome (i.e., local progression-free survival and OS), a biologically equivalent dose ≥ 65 Gy was calculated to treat unresectable disease [13]. The average total dose of our investigation was 64.3 Gy, reflecting 56.6% adjuvantly treated cases. The median total dose for adjuvant setting at MSKCC was 63 Gy [15]. The group of Gabriele et al. prescribed for adjuvant RT 60 Gy median total dose to the PTV and 68 Gy for definitive RT. In all cases, RT application was 3D-conformal, resulting in a 5 year local control rate of 74% (adjuvant) and 20% (definitive) with a 5 year OS rate of 72% vs. 25% [32]. Approximately two-thirds of MSKCC-patients had CT-based treatment planning and 3D-conformal or IMRT [15,33]. Only a few cases were treated by 2D-RT.

We did not investigate the role that histological types might play concerning five-year composite OS because of the small number of histologies other than SCC. Other histological types were rather rare and could not be expected to lead to statistically powerful analyses. In turn, we were aware that survival analysis of all tumor histologies in totality could lead to a decrease concerning five-year composite overall survival. This effect was partially seen when comparing our data to published data [2,6,16]. We hypothesize the cumulative effect of different histologies, and therefore, our five-year composite OS observations correlate with published data.

As is well-known, the diagnosis and therapy of sinonasal tumors is challenging for the multidisciplinary team in finding the best way to cure the patient while maintaining the lowest achievable amount of side effects. In addition, in the very early stages, these tumors produce nonspecific inflammatory-like symptoms [22]. Unfortunately, this leads to a delay in diagnosis and, subsequently, the presence of advanced stages. We suspect such delays in the diagnosis of our patients because of the large majority of patients with advanced tumor stages III—IVB (82% of all cases) we have observed.

In our study, 56.6% of the patients were treated with adjuvant RT. 35.8% of the patients received definitive RT, in some cases with platinum-based chemotherapy. Almost half of all patients (43.4%) received RCT. The comparison of RT coupled with RCT did not confirm a benefit through the addition of chemotherapy but showed a lower 5 year composite OS rate for RCT (45.8 ± 9.8% vs. 38.5 ± 12.6%, *p* = 0.62) (Figure 4). This result may be due to chemotherapy indications. Only patients at high risk with certain tumor hallmarks, such as a primary tumor in situ, positive resection margins or extracapsular nodal spread, received concurrent chemotherapy.

Here, we compared two boost concepts of IMRT for sinonasal carcinoma using photons. The SIB patients reached a 5 year OS of 51.8% compared to 23.3% after SEQ, although this is only a trend without statistical significance (*p* = 0.177). We speculate that a “light” dose escalation using SIB (2.2 Gy to 70.4 Gy for definitive RT and 2.14 Gy to 64.2 Gy for adjuvant RT) in particular for primary tumors could yield higher biological equivalent doses (BED) using a higher single dose and a reduction of treatment time, as it was proposed that only a small number of tumor cells have a self-renewal capacity and could be responsible for tumor relapse [34]. It may also be supposed that eliminating sinonasal tumors containing such cells would require a local dose escalation. Nevertheless, further investigations of this issue are needed.

Another promising survival predictor described in the literature was GTV for patients with oropharyngeal carcinomas (OPC). The DKTK-ROG study showed, among others, a linear correlation between GTV and a 2-year locoregional control is primarily treated, locally advanced OPC [35]. GTV as an independent overall prognostic survival factor was also shown in another study on definitively treated p16-negative OPC [36]. Local control and GTV were shown to be positively correlated in advanced head and neck squamous cell carcinoma (HNSCC) as well [37]. Little data exist for the correlation of pre-therapeutic GTV and the survival of patients with sinonasal tumors. Patients with a pre-therapeutic GTV smaller than 75 cm^3^ reached a significantly better five-year composite OS, independent of the RT setting. We found that a pre-therapeutic GTV larger than 100 cm^3^ is a predictive factor for worse composite OS for adjuvant and definitive RT. Patients after surgery and RT with small initial tumor volumes reached the best outcome, followed by the RT/RCT group with small tumor volumes. Patients with initial tumors larger than 100 cm^3^ did not benefit from the combination of surgery and RT. Their composite OS was comparable to that of the group that only received RT (5 year composite OS adjuvant 25 ± 20.4% vs. definitive 29.8 ± 13.3%). In our research, pre-therapeutic GTV are large compared to data from literature [35,36,37,38,39,40]. The reported pre-therapeutic tumor volumes range between mean GTV 26.6 ± 21.2 cm^3^ [38] defined by T1 contrast-enhanced MRI, mean GTV 12.79 ± 24.31 cm^3^ [39] defined by all MRI sequences, and median GTV-only tumor 73.4 cm^3^ [43.0–119.5] [40] defined by MRI and CT. Hennersdorf et al. reported a significantly better progression-free survival (PFS) of 63 months with tumor volumes < 25.4 cm^3^ versus 38.7 months (*p* = 0.004) [38]. Eley et al. showed a correlation between tumor volume, OS, and disease-free survival (DFS). This significant difference diminished after five years [39]. The group of Ferella et al. investigated in detail the GTV of the primary tumor, the cervical lymph nodes, and the retropharyngeal lymph nodes. Only 34 SCC were included. In summary, they discovered an influence of GTV-only tumor on the OS and PFS. If the total GTV, including all macroscopic involved lymph nodes, was larger than 149.44 cm^3^, OS and PFS were reduced compared to lower total tumor volumes [40]. The initial pre-surgical macroscopic GTV of our patients was contoured on pre-therapeutic imaging (MRI or CT or both) for postoperative target delineation in the RT planning system. In the present study, we clearly show that initial GTV is a prognostic factor for the five-year composite OS in sinonasal carcinoma, as shown by other investigators. If a patient does not benefit from combined modality treatment (surgery and RT) due to a large tumor volume, the treatment strategy should be discussed in detail between the treatment teams and the patient. The impact of the pre-therapeutic GTV on the composite OS should be factored into the treatment decision. Surgery could be omitted in such cases by treating the patient following the best therapeutic ratio by securing the least damage possible by way of therapy.

As in all other publications in the field of sinonasal carcinoma, we battle with the limitation of a small retrospective patient cohort and various histologies. A rare tumor entity with small subgroup numbers is a drawback for clear therapy recommendations, especially when attempting to assess the impact of pre-therapeutic GTV on the five-year composite OS while having to consider different histologies. Consequently, statistics are limited, and results should be interpreted with caution.

## 5. Conclusions

The pre-therapeutic GTV is a prognostic factor for composite OS in patients with sinonasal tumors. It influences the outcome after all treatment strategies. Small sinonasal tumors should be removed and treated adjuvantly by RT to achieve the best composite OS. Patients with larger tumor volumes do not benefit from surgery combined with RT. In these particular cases, an intensive, multidisciplinary discussion is necessary to obtain the best treatment option for the patient and to thereafter communicate these facts honestly with the patient in the process of shared decision-making. Against the background of the presented results omitting extensive traumatic surgery, radiooncological treatment alone is a good alternative to retain the quality of life by the comparable outcome.

The simultaneous integrated boost (SIB) technique seems to improve OS but failed to reach statistical significance over SEQ while yielding better OAR sparing.

## Figures and Tables

**Table 1:** Patient characteristics (*n* = 53).

Characteristic	Number of Patients	Percentage
Sex		
Male	35	66%
Female	18	34%
Age at diagnosis (years)		
Median (min–max)	60	(35–86)
Localization		
Nasal cavity	11	20.8%
Paranasal sinus	27	50.9%
Both localizations	15	28.3%
Tumor histology		
Squamous cell carcinoma	32	60.4%
Adenocarcinoma	8	15.1%
Adenoid cystic carcinoma	4	7.5%
Mucoepidermoid carcinoma	3	5.7%
Undifferentiated carcinoma (SNUC)	3	5.7%
Esthesioneuroblastoma	2	3.8%
Carcinoma ex pleomorphic adenoma	1	1.9%
Disease stage (AJCC staging system, 2010)		
I	0	0%
II	9	17.0%
III	11	20.8%
IVA	23	43.4%
IVB	10	18.9%

**Table 2:** Patient treatment characteristics (*n* = 53).

Parameter	Number of Each Parameter	Percentage
Therapy intention		
Adjuvant	30	56.6%
Definitive	19	35.8%
Additive (postop. R+)	2	3.8%
Early recurrence	1	1.9%
Palliative	1	1.9%
Concurrent chemotherapy		
None	30	56.6%
Cisplatin 40 mg/m^2^ qd7	15	28.3%
or Carboplatin AUC 2 qd7		
Cisplatin 20 mg/m^2^ week 1 + 5	5	9.4%
Cisplatin 100 mg/m^2^ qd21	3	5.7%
RT Technique		
2D/3D	4	
IMRT/VMAT	18	
HT	31	
Boost concept		
SEQ	25	
SIB	27	
Both	1	
Duration of RT (d)		
Mean (min–max)	43	(20–53)
Single dose (Gy)		
Mean (min–max)	2.0	(1.7–3.0)
Total dose (Gy)		
Average (min–max)	64.3	(45–74)

## Data Availability

The datasets used and/or analyzed during the current study are available on demand from the authors.
